# Conservative Restoration of Bone Without Casting or Surgery for Charcot Foot in a Diabetic Patient

**DOI:** 10.1002/ccr3.70330

**Published:** 2025-03-14

**Authors:** Mutasem Iqnaibi, Issa Kh. Aldababseh, Kareem lbraheem, Omama Dababsah, Raef Sabra, Ruaa Adwan

**Affiliations:** ^1^ Faculty of Medicine Palestine Polytechnic University Hebron Palestine; ^2^ Anaesthesia Department Alia Governmental Hospital Hebron Palestine

**Keywords:** charcot neuroarthropathy, conservative management, diabetes, diabetic foot ulcer, ozonated saline therapy

## Abstract

This case highlights the successful management of a 52‐year‐old male with poorly controlled diabetes and Charcot neuroarthropathy. Early diagnosis and a holistic treatment plan, including antibiotics, vitamins, and local interventions, improved the patient's condition, preventing amputation. Emphasis on glycemic control and patient education is critical for effective management.

## 1 Introduction

Charcot disease, also called Charcot neuroarthropathy, is a progressive illness that mainly affects patients with peripheral neuropathy, which is frequently brought on by diabetes mellitus [1]. It affects the bones, joints, and soft tissues of the feet and ankles. Inflammation, fractures, and deformities are symptoms of the disease that, if left untreated, can cause severe disability [2]. Early diagnosis, immobilization of the affected limb, strict glucose control, and infection prevention are all stressed in standard treatment protocols [1, 3]. Pharmacological interventions, offloading techniques, and surgical correction are available as treatment options in more severe cases [3].

Even though the management of Charcot disease has certain well‐established fundamentals, there are still many unanswered questions about the most effective treatment combinations, particularly for acute presentations and preventing long‐term complications. It is also unclear to what extent novel treatments, such as ozonated saline, might contribute to management strategies, given their antimicrobial and anti‐inflammatory properties [4, 5]. This report aims to address these gaps by presenting a case that highlights the potential of conservative management using ozonated saline, alongside a comprehensive therapeutic regimen, to avoid surgical intervention and improve patient outcomes.

## 2 Case History/Examination

**FIGURE 1:**
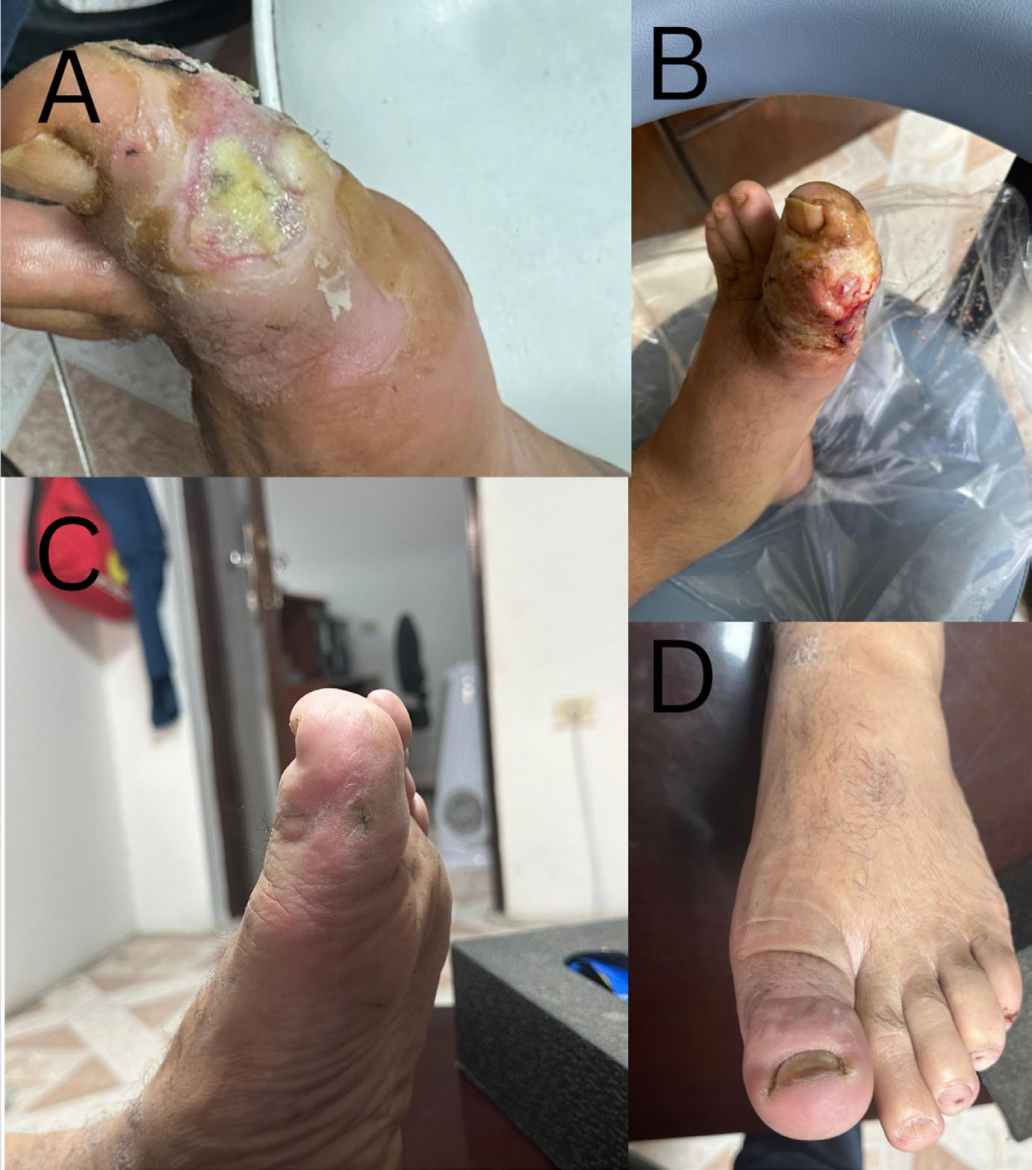
(A, B) The left big toe appears to be swollen and erythematous with yellowish discharge. (C, D) The left big toe appears healthy, and healed. Without redness or swelling.

A 52‐year‐old male with a history of poorly controlled diabetes mellitus presented to the clinic with a chief complaint of a skin wound on his right foot, accompanied by cellulitis. Initial laboratory results showed an HbA1c level of 12.9%, indicating severe hyperglycemia, and a C‐reactive protein (CRP) level of 120 mg/L, suggesting systemic inflammation. The patient had been managing his diabetes poorly, consuming approximately 2 L of juice daily, and had returned to the clinic two months later with a spontaneous fracture of his right second toe. On examination, the patient exhibited cellulitis, swelling, and a painless open wound with yellowish discharge on the left big toe (Figure 1). Additional history revealed limited prior diabetes education and the use of sitagliptin/metformin (50/1000 mg) and metformin (850 mg), with Lantus insulin added as needed during the treatment period.

### 2.1 Investigations

**FIGURE 2:**
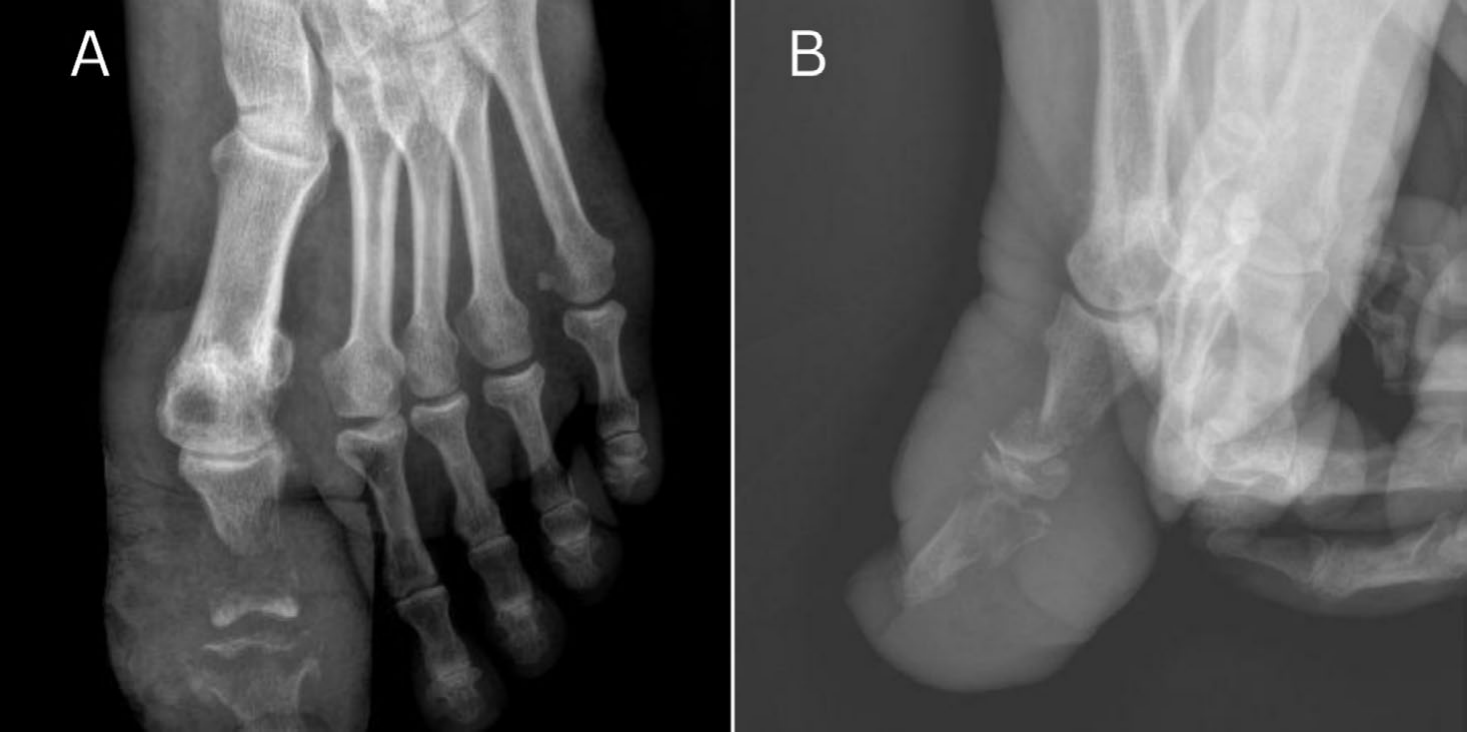
Pre‐treatment (A) X‐ray: Bony rarefaction in the distal part of the proximal phalanx of the big toe. It is associated with soft tissue swelling. (B) X‐ray: Progression of the disease is seen with rarefaction of the distal part of the proximal phalanx as well as the proximal part of the distal phalanx. Soft tissue swelling is still seen even to more extent than it was noted in right X‐ray.

**FIGURE 3:**
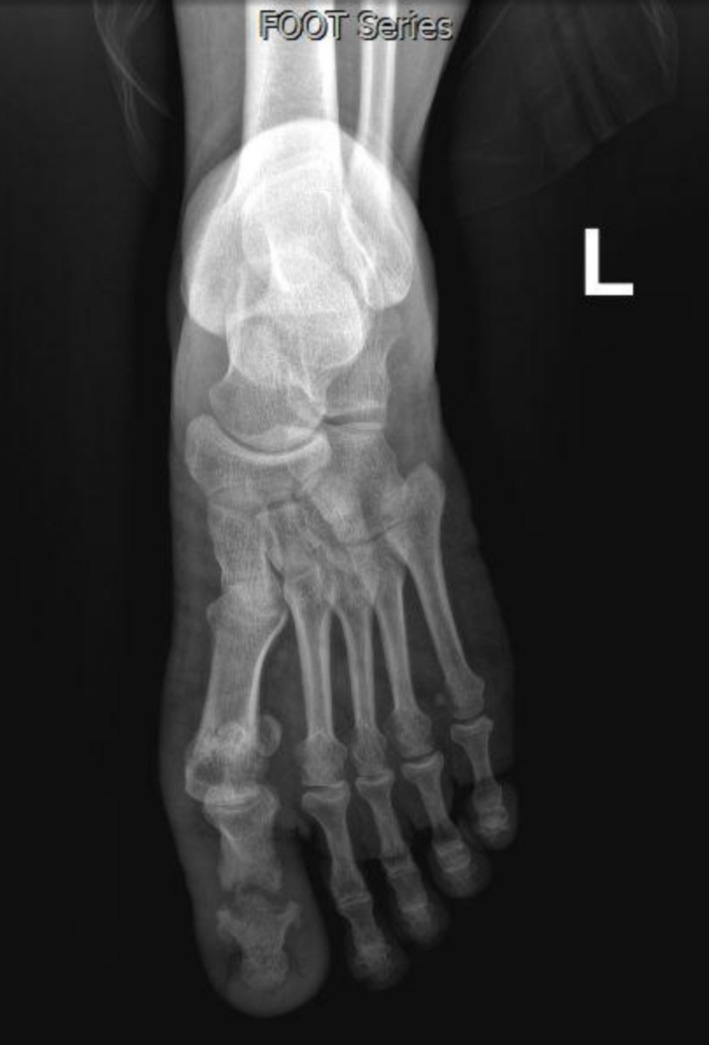
During treatment X‐ray: The soft tissue swelling is seen less with destruction and disorganization of the inter‐phalangeal joint (Charcot joint).

Wound cultures: No bacterial growth. Radiological imaging: Initial findings: Bony rarefaction in the distal part of the proximal phalanx of the big toe, with soft tissue swelling (Figure 2A,B). Follow‐up imaging: Progression of rarefaction in the proximal and distal phalanx and increased soft tissue swelling (Figure 3). The patient's Foot and Ankle Outcome Score (FAOS) was 285/500 points; it indicates a moderate level of impairment (FAOS symptoms: 75; FAOS pain: 60; FAOS activities of daily living: 55; FAOS sports and recreation: 55; and FAOS quality of life: 40).

## 3 Differential Diagnosis

The differential diagnosis focused on three main possibilities. Charcot neuroarthropathy was the leading consideration due to the presence of a deformed, swollen foot with progressive radiographic changes and the absence of significant pain, consistent with the neuropathic nature of the condition. Osteomyelitis was another key possibility, given the chronic wound and associated soft tissue changes; though the lack of systemic signs of infection reduced its likelihood. Lastly, septic arthritis was considered, but the clinical presentation and imaging findings, including the absence of joint effusion, favored Charcot neuroarthropathy over infectious causes.

## 4 Conclusion and Results

Initial management included oral antibiotics and local application of ozonated saline every two days for three weeks, which led to symptom improvement. After the diagnosis of Charcot foot: Vitamin K2, vitamin D3 (5000 IU daily), calcium carbonate (1200 mg daily), and a multivitamin were prescribed. Aspirin (100 mg three times daily) was added to improve circulation and address ischemia. Two lateral incisions were made at the fracture site to facilitate drainage of purulent discharge, and the area was irrigated with ozonated saline. The patient engaged in pool water walking and swimming exercises to improve mobility and reduce stress on the affected foot. Over two months, he adjusted his weight‐bearing to walking on his heel and used a bicycle for mobility.

**FIGURE 4:**
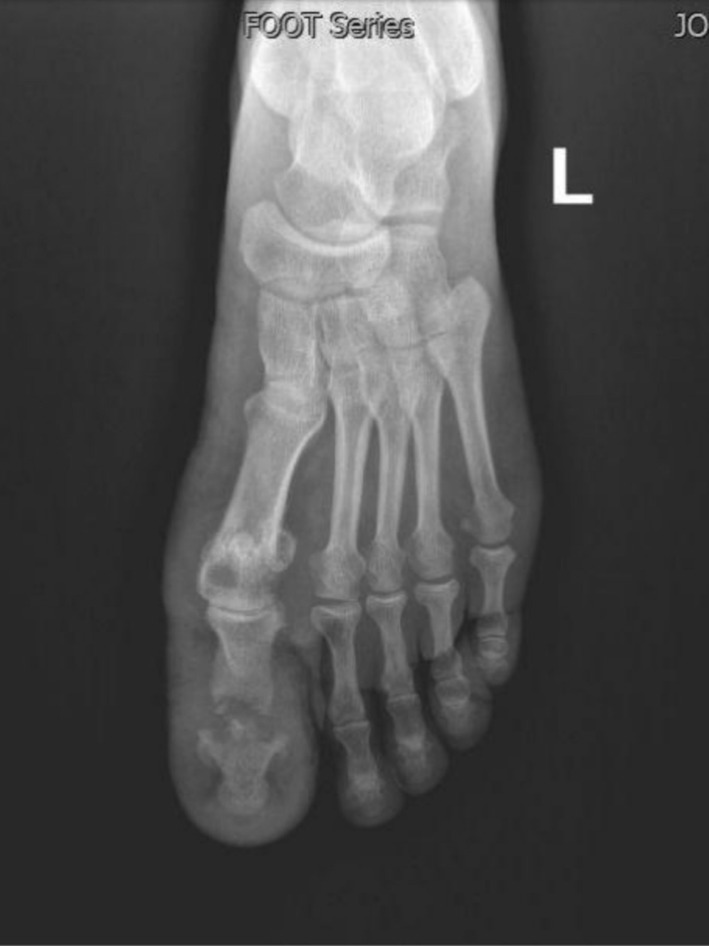
Post‐treatment X‐ray: The soft tissue swelling has subsided. Better mineralization of the bones is noted.

After the initial treatment, the patient demonstrated clinical improvement. However, due to poor glycemic control, he experienced a spontaneous fracture and worsening of Charcot foot symptoms. Ongoing treatment resulted in a significant reduction in wound discharge and improvement in local tissue health. Subsequent imaging revealed decreased soft tissue swelling but continued destruction and disorganization of the interphalangeal joint. With consistent treatment, there was a significant reduction in wound discharge and improvement in local tissue health (Figure 4). However, the progression of bone destruction and joint disorganization despite conservative management raised concerns about the sufficiency of this approach. Surgical intervention was avoided due to the patient's poor glycemic control and systemic risks. Imaging revealed improved bone mineralization but highlighted the limitations of non‐surgical approaches in addressing advanced bone destruction. Long‐term management emphasized comprehensive diabetes control and continued mobility adjustments without casting, alongside vitamin supplementation and antibiotics to prevent further complications.

In conclusion, this case highlights the successful conservative restoration of bone integrity in a patient with Charcot foot, achieved without casting or surgery. Early recognition of Charcot neuroarthropathy, coupled with an approach emphasizing infection control, wound care, and bone health, played a critical role in preventing severe complications. This case underscores the importance of individualized care strategies tailored to the specific needs of diabetic patients with complex complications.

## 5 Discussion

Charcot disease in diabetic patients presents a significant clinical challenge due to its complex pathophysiology and potential for severe complications, including limb amputation [6]. This case underscores the importance of early diagnosis and a comprehensive treatment approach in managing Charcot disease.

The initial presentation of our patient, with a high HbA1c level of 12.9% and elevated CRP, indicates poor diabetes control and systemic inflammation—both contributing factors to the development of Charcot neuroarthropathy [7]. These findings highlight the critical role of addressing hyperglycemia and systemic inflammation early in the disease course. The use of ozonated saline demonstrated a promising initial response, supporting its potential to reduce inflammation and promote wound healing [5].

However, the progression of bone destruction despite these interventions raises questions about the sufficiency of conservative management. This limitation underscores the need for a balanced consideration of conservative versus surgical interventions, especially in advanced cases. The absence of surgical intervention in this case was dictated by systemic risks associated with the patient's poor glycemic control. However, the replicability of this approach across different settings or patient populations remains uncertain, as access to ozonated saline and specialized care may not be universally available. Future studies are needed to establish evidence‐based guidelines that consider these limitations [8].

Charcot disease often appears unexpectedly and can quickly progress into a severe, permanent foot deformity, leading to ulceration and amputation. Severe deformities and damage to the foot and ankle result from an uncontrolled cycle of inflammation [9]. The patient's subsequent neglect of diabetes management, which led to hyperglycemia and further complications, emphasizes the critical role of glycemic control in preventing the progression of Charcot disease. Poor glycemic control exacerbates neuropathy and impairs wound healing, creating a vicious cycle that complicates the management of Charcot neuroarthropathy [10]. This case highlights the need for continuous patient education and monitoring to ensure adherence to diabetes management protocols.

In this case, educational interventions and continuous monitoring were employed to improve adherence, but the patient's subsequent neglect of diabetes management underscores the persistent difficulty in maintaining long‐term compliance. Structured diabetes education programs, involving both patients and caregivers, along with the use of technology such as glucose monitoring devices, may enhance adherence and glycemic control.

The gold standard for treating Charcot neuroarthropathy (CN) is non‐operative therapy with a total contact cast, followed by proper bracing and footwear. When conservative treatment proves ineffective, surgery becomes necessary. Immobilization and protected weight bearing are effective treatments for the majority of CN patients [9].

The comprehensive treatment regimen, including high doses of vitamin K2, vitamin D, calcium, multivitamins, and aspirin, aimed to address the multifactorial aspects of Charcot disease. Vitamin D and calcium are essential for bone health and repair [11] while vitamin K2 is crucial for bone mineralization and preventing vascular calcification [12]. The use of aspirin to improve circulation and address ischemia associated with Charcot disease is supported by its known benefits in enhancing blood flow and reducing inflammation [13].

Ozonated saline was selected for its antimicrobial and tissue‐regenerative properties, as supported by clinical studies [3, 14, 15, 16, 17]. The decision to perform lateral incisions and flush the fracture site with ozonated saline was pivotal in managing the ongoing discharge and promoting bone regeneration [3]. According to Kushmakov et al., local ozone therapy may shorten the course of treatment and reduce the risk of infection. However, regarding the disadvantages of ozone therapy, such as ozone gas toxicity, its therapeutic utility depends on the concentration, the site of administration, and the treatment type [18]. Ozone treatment was used to prevent a 67‐year‐old lady with a diabetic foot from getting her foot amputated in a case study by Aytacoglu et al. [19].

This intervention aligns with the principles of surgical debridement and infection control, which are essential in managing osteomyelitis and chronic wounds in Charcot disease. The successful outcome observed in this case, including significant bone regeneration and the avoidance of amputation, supports the efficacy of this approach [16]. The intensification of treatment, including increased doses of ozone, nutrients, and aspirin, alongside the administration of probiotics and targeted therapy for urinary tract infection, highlights the importance of a holistic approach in managing Charcot disease. Probiotics have been shown to modulate the immune response and improve gastrointestinal health, which can be beneficial in patients with chronic diseases and those receiving long‐term antibiotic therapy [14, 15].

This case underscores the potential of an aggressive, multidisciplinary approach in managing severe Charcot disease and avoiding limb amputation [17]. However, it is essential to address whether this approach is replicable across different settings or populations. Factors such as access to specialized care, patient compliance, and the availability of ozonated saline or advanced therapies may limit the generalizability of this case. Future studies should explore scalable and standardized approaches to managing Charcot disease while ensuring that individual patient factors are accounted for. Furthermore, long‐term outcomes and strategies to prevent complications, such as structured rehabilitation programs and integrated care models, should be emphasized to improve overall management and patient quality of life.

## 6 Conclusion

This case highlights the critical importance of early diagnosis, effective glycemic control, and a comprehensive therapeutic approach in managing Charcot disease. Conservative management with ozonated saline demonstrated potential benefits, reducing inflammation and promoting healing. However, the limitations of this approach, particularly regarding continued bone destruction, underscore the need for ongoing research and patient education to refine treatment protocols and improve outcomes. Translating these findings into broader clinical practice will require further validation and exploration of long‐term impacts.

## Author Contributions

**Mutasem Iqnaibi:** conceptualization, data curation, investigation, supervision, validation, writing – original draft. **Issa Kh. Aldababseh:** conceptualization, data curation, investigation, writing – original draft, writing – review and editing. **Kareem lbraheem:** conceptualization, data curation, formal analysis, resources, software, writing – original draft, writing – review and editing. **Omama Dababsah:** data curation, formal analysis, methodology, resources, writing – original draft. **Raef Sabra:** data curation, formal analysis, writing – review and editing. **Ruaa Adwan:** data curation, formal analysis, investigation, writing – review and editing.

## Consent

Written informed consent was obtained from the patient for the publication of this case report.

## Conflicts of Interest

The authors declare no conflicts of interest.

## Data Availability

The data used to support the findings of this study are included in the article.
